# Supplementary material for: Identification of essential genes for Escherichia coli aryl polyene biosynthesis and function in biofilm formation
Source: NPJ Biofilms Microbiomes. 2021 Jul 2;7:56. doi: 10.1038/s41522-021-00226-3 (PMC8253772; doi:10.1038/s41522-021-00226-3)
Supplement: Supplementary file 1 — Supplementary Information [file 41522_2021_226_MOESM1_ESM.pdf]

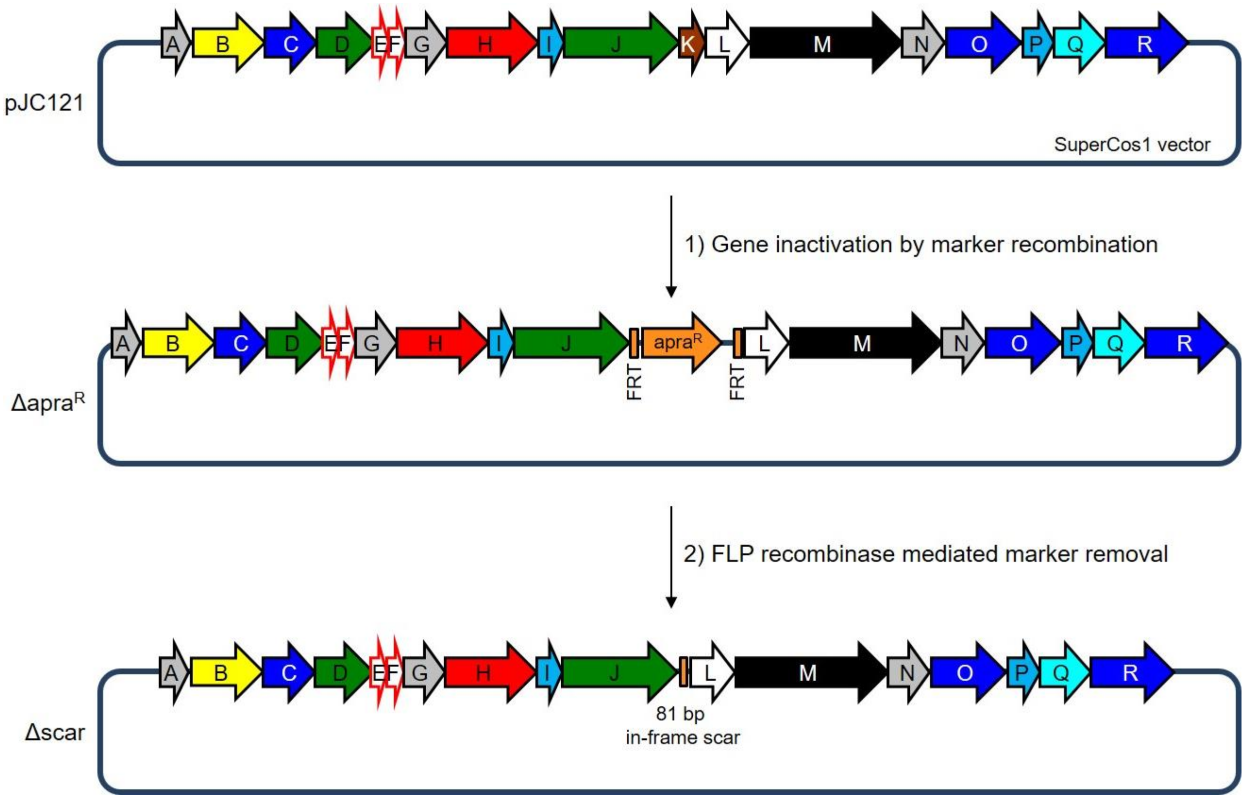

Supplementary Figure 1. Schematic overview of scar mutagenesis procedure. Individual genes in the APE_{Ec} BGC were replaced with an 81 bp in-frame scar sequence in a two-step PCR-generated recombineering process[28]. This is exemplified by *apeK* deletion, other genes were replaced in an analogous manner. **Step 1)** The pIJ121 parent vector and a gene-specific targeting cassette are introduced into an *E. coli* strain expressing the λRED recombination machinery, and the gene of interest is replaced with the $apra^R$ marker via double homologous recombination, yielding $\Delta apra^R$ plasmids for each gene (even numbered plasmids pJC130-164 in Table S3). **Step 2)** The resistance marker is removed via Flp mediated recombination of the $apra^R$ flanking FRT sites, yielding in-frame Δscar plasmids (odd numbered plasmids PJC131-165 in Table S3).

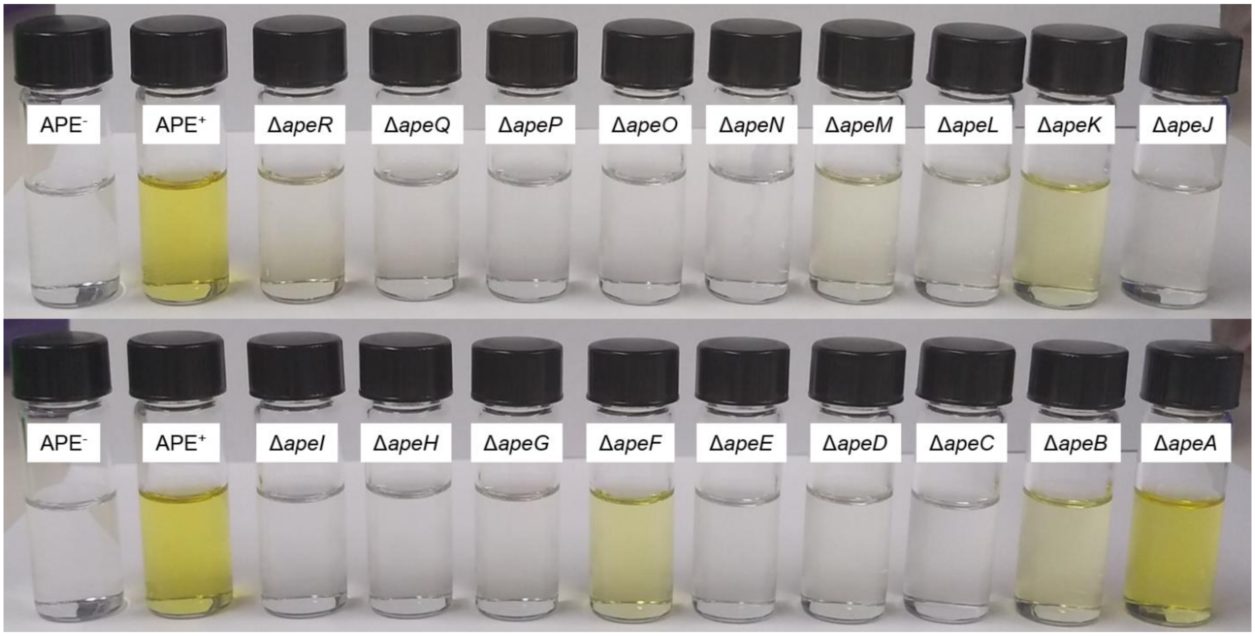

Supplementary Figure 2. Crude cell extracts of the different *E. coli* Δ*ape* strains. Cell pellets from the APE^-, APE^+ and various Δ*ape* mutants (Fig. 2) were extracted, filtered over paper, and evaporated to dryness prior to reconstitution in MeOH.

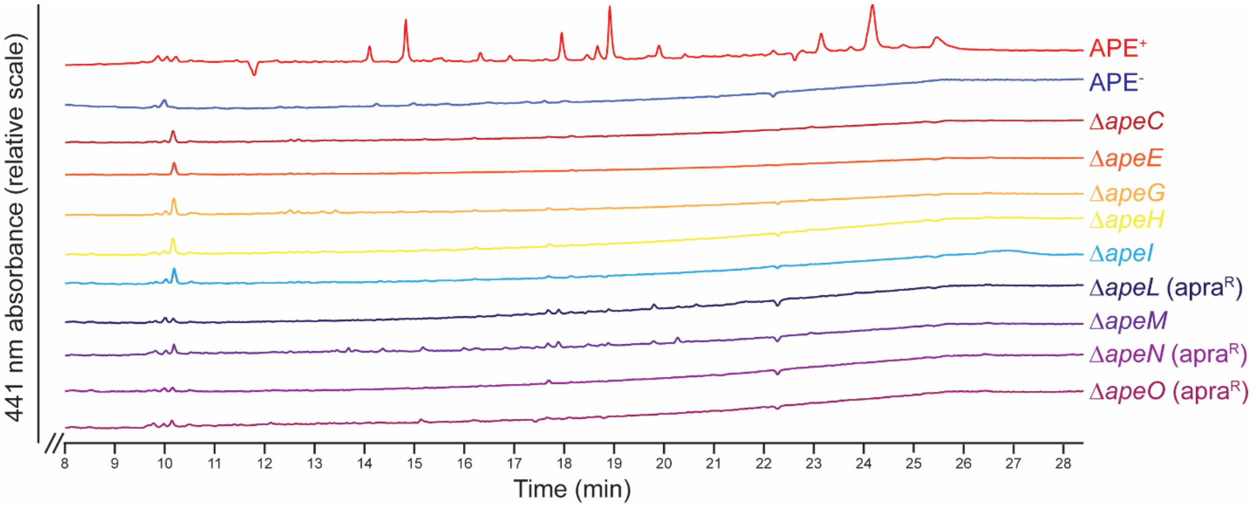

Supplementary Figure 3. HPLC traces of *E. coli* Δ*ape* strain crude extracts that did not have significant 441 nm absorbance. These strains comprise mostly the genes involved in biosynthesis of the core APE_{Ec}-carboxylic acid moiety (*apeC*, *apeE*, *apeHI* and *apeO*), as well as some genes predicted to be involved in transport (*apeG* and *apeLMN*). The APE^{+} extract (red) and APE^{-} strain (dark blue) are reproduced here from Fig. 4 for reference. Samples were run under the same HPLC conditions (a gradient of ACN in 0.1% trifluoroacetic acid (TFA) water, initiating with a hold at 100% water for 3 min, followed by gradual increase from 0% to 100% ACN between 3 min and 23 min, and ending in a 100% ACN hold). Detection was at λ = 441nm.

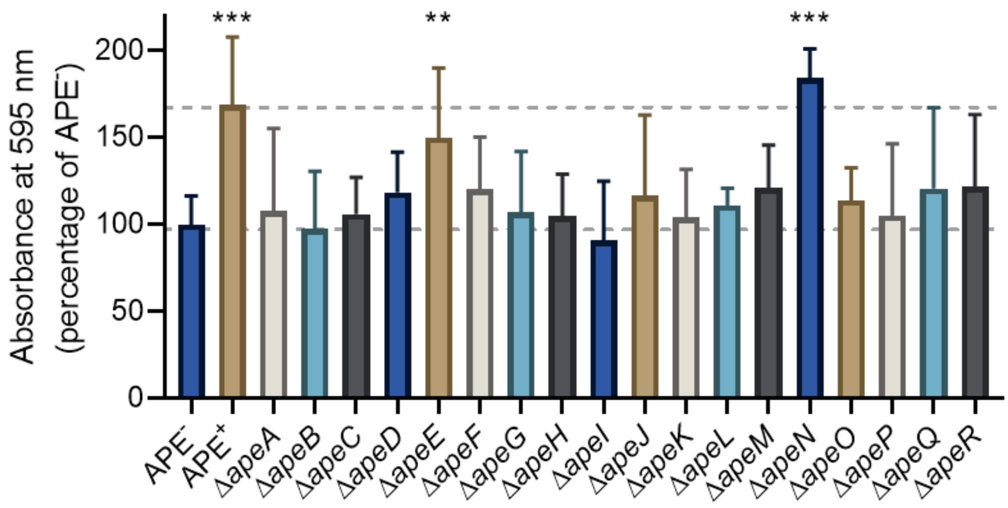

Supplementary Figure 4. Crystal violet quantification of biofilms formed by the different Δ*ape* strains. The APE^+, Δ*apeE* and Δ*apeN* strains showed a significant increase in biofilm formation compared to APE^-. Biofilms were grown in non-treated multi-well plates with MH medium for 48 hours and subsequently stained with crystal violet. Absorbance at 595 nm was measured and normalized to APE^- values (n=3 repeats; error bars represent SD; $^{**}p<0.01$, $^{***}p<0.001$ by unpaired, two-tailed t-test).

Supplementary Table 1. Strains used in this study

Strain	Description	Reference
CFT073	uropathogenic *E. coli* strain, serotype O6:H1:K2	[54]
Top10	*E. coli* cloning strain	Invitrogen
BW25113	*E. coli* K-12 derivative: Δ*araBAD*, Δ*rhaBAD*	[55]
BT340	*E. coli* DH5α/pCP20	[56]

Supplementary Table 2. Primers used in this study

Primer	Sequence (5'→3')
apeA F	AATTATATAATTGTATTGCATACATAAGAGGCACTAATGATTCCGGGGATCCGTCGACC
apeA R	ATACTATCGGGCCATTCCAGCCCGATATCAGCATCACTATGTAGGCTGGAGCTGCTTC
apeB F	GCCATCACCGAAGCACAGCGGATTGCCTTTGCTCCTATGATTCCGGGGATCCGTCGACC
apeB R	GCGCGCCCGCGGTAAACCAAAATTATTAAGTGACGATTATGTAGGCTGGAGCTGCTTC
apeC F	CACTTAATAATTTTGGTTTACCGCGGGCGCGCGTTGATGATTCCGGGGATCCGTCGACC
apeC R	TCATCTGCGGCTCCAGCGCCATTGCACGCGCTCGCCAGGTGTAGGCTGGAGCTGCTTC
apeD F	CGTGCAATGGCGCTGGAGCCGCAGATGAGTAAGCTGATGATTCCGGGGATCCGTCGACC
apeD T	TTAATTTCCAGATAAAGCGCTTGCATTATTTATTCCTGATGTAGGCTGGAGCTGCTTC
apeE F	TAAATAATGCAAGCGCTTTATCTGGAAATTAAAAACCTCATTCCGGGGATCCGTCGACC
apeE R	TGGTTTGTTGGTCTGTCATGATGGTTTTCCTCAGGCACGTGTAGGCTGGAGCTGCTTC
apeF F	TTTCATTGCTGCGCAACGTGCCTGAGGAAAACCATCATGATTCCGGGGATCCGTCGACC
apeF R	AGCGAGCGAATGCCCGACATCAAATTAGCCTTCTTGTAGTGTAGGCTGGAGCTGCTTC
apeG F	AGGCTGTAGAACGCCTGCTACAAGAAGGCTAATTTGATGATTCCGGGGATCCGTCGACC
apeG R	GTGAAAGAGAGAGAGTCTGATTCATGGTGTTTTCCGTTTTGTAGGCTGGAGCTGCTTC
apeH F	ACGTCGTAAGATGATCAAACGGAAAACACCATGAATCAGATTCCGGGGATCCGTCGACC
apeH R	CATGAAATAACTCCTGTAATTGCGCATAGACACGCTTATTGTAGGCTGGAGCTGCTTC
apeI F	AATTACAGGAGTTATTTCATGATACGCCATGAAATTGAGATTCCGGGGATCCGTCGACC
apeI R	GGTTGTAGCAGGGGATCAACACGCAGGGAGAAAAGTTTATGTAGGCTGGAGCTGCTTC
apeJ F	GCGATGATGCCGGGCGTGCTGGCGCGTCTTAAGCCATTTATTCCGGGGATCCGTCGACC
apeJ R	GGTAAATCGGGGATCGTTAAGCACCCTTTACTCCTTGTCTGTAGGCTGGAGCTGCTTC
apeK F	TGGCAACTGCCGGAAATTCAGGACAAGGAGTAAAGGGTGATTCCGGGGATCCGTCGACC
apeK R	GCGCCAGCAGCGGTAAAAATTTCATGGTTTGACTCCCATTGTAGGCTGGAGCTGCTTC
apeL F	TATTCTGTTTGAACGCATGGGAGTCAAACCATGAAATTTATTCCGGGGATCCGTCGACC
apeL R	GGCAAAACGTTGGTGTTCGTCATCGGTCAGTTGCGCTGGTGTAGGCTGGAGCTGCTTC
apeM F	CCGATGACGAACACCAACGTTTTGCCGCCCAGTAAACGCATTCCGGGGATCCGTCGACC
apeM R	CTCGCCAGAATGTGGATTTGATCATTTTTTTGTTCTCTTTGTAGGCTGGAGCTGCTTC
apeN F	GCTGGCGATGCCCGATAAAAAGAGAACAAAAAAATGATCATTCCGGGGATCCGTCGACC
apeN R	GCGGAAATATAAATCATATCAGTCACCTAAGTATTGAATTGTAGGCTGGAGCTGCTTC
apeO F	AATACCACATCACCATTCAATACTTAGGTGACTGATATGATTCCGGGGATCCGTCGACC
apeO R	CGCCGGGGGATAAATAGTGGCTCACGAAACCCTCCCGAGTGTAGGCTGGAGCTGCTTC
apeP F	TAACGCCAGCATTCTGCTCGGGAGGGTTTCGTGAGCCACATTCCGGGGATCCGTCGACC
apeP R	TAACCAGAACTGAACGACTCATCAGGACGCTCCTTGTTGTGTAGGCTGGAGCTGCTTC
apeQ F	ACTTACCACCCTGTTTCAACAAGGAGCGTCCTGATGAGTATTCCGGGGATCCGTCGACC
apeQ R	TAATCACTACGCGACGTGTCATAACATCCCTCCATTGATTGTAGGCTGGAGCTGCTTC
apeR F	CCGCCAGGTTATTTCCATCAATGGAGGGATGTTATGACAATTCCGGGGATCCGTCGACC
apeR R	GAATCACCACATGGGATGTCCATGTGGTTGTATAACTCATGTAGGCTGGAGCTGCTTC

Supplementary Table 3. Plasmids used in this study

Plasmid	Description	Reference
SuperCos1	cosmid vector (kan^R, apra^R)	Agilent Inc.
pIJ773	pBS SK+ with cassette P1-FRT-*oriT*-*aac(3)IV*-FRT-P2	[28]
pIJ790	λ-RED (*gam*, *bet*, *exo*), *cat*, *araC*, rep101ts	[28]
pJC121	SuperCosI::CFT073-ape cluster (c1186-c1204)	[1]
pJC130	pJC121 Δ*apeA*::apraR	present study
pJC131	pJC121 Δ*apeA*::scar	present study
pJC132	pJC121 Δ*apeB*::apraR	present study
pJC133	pJC121 Δ*apeB*::scar	present study
pJC134	pJC121 Δ*apeC*::apraR	present study
pJC135	pJC121 Δ*apeC*::scar	present study
pJC136	pJC121 Δ*apeD*::apraR	present study
pJC137	pJC121 Δ*apeD*::scar	present study
pJC138	pJC121 Δ*apeE*::apraR	present study
pJC139	pJC121 Δ*apeE*::scar	present study
pJC140	pJC121 Δ*apeF*::apraR	present study
pJC141	pJC121 Δ*apeF*::scar	present study
pJC142	pJC121 Δ*apeG*::apraR	present study
pJC143	pJC121 Δ*apeG*::scar	present study
pJC144	pJC121 Δ*apeH*::apraR	present study
pJC145	pJC121 Δ*apeH*::scar	present study
pJC146	pJC121 Δ*apeI*::apraR	present study
pJC147	pJC121 Δ*apeI*::scar	present study
pJC148	pJC121 Δ*apeJ*::apraR	present study
pJC149	pJC121 Δ*apeJ*::scar	present study
pJC150	pJC121 Δ*apeK*::apraR	present study
pJC151	pJC121 Δ*apeK*::scar	present study
pJC152	pJC121 Δ*apeL*::apraR	present study
pJC154	pJC121 Δ*apeM*::apraR	present study
pJC155	pJC121 Δ*apeM*::scar	present study
pJC156	pJC121 Δ*apeN*::apraR	present study
pJC158	pJC121 Δ*apeO*::apraR	present study
pJC160	pJC121 Δ*apeP*::apraR	present study
pJC161	pJC121 Δ*apeP*::scar	present study
pJC162	pJC121 Δ*apeQ*::apraR	present study
pJC163	pJC121 Δ*apeQ*::scar	present study
pJC164	pJC121 Δ*apeR*::apraR	present study
pJC165	pJC121 Δ*apeR*::scar	present study
